# Domestic violence and its relationship with depression, anxiety and quality of life: A hidden dilemma of Pakistani women

**DOI:** 10.12669/pjms.37.1.2893

**Published:** 2021

**Authors:** Mazhar Malik, Nargis Munir, M. Usman Ghani, Nasir Ahmad

**Affiliations:** 1 Prof. Dr. Mazhar Malik, Professor of Psychiatry, HoD Department, Department of Psychiatry and Behavioural Sciences, Rawal Institute of Health Sciences, Rawal General and Dental Hospital, Lehtrar Road, Islamabad, Pakistan; 2 Nargis Munir, Clinical Psychologist, Department of Psychiatry and Behavioural Sciences, Rawal Institute of Health Sciences, Rawal General and Dental Hospital, Lehtrar Road, Islamabad, Pakistan; 3 Dr. M. Usman Ghani, Assistant Professor of Psychiatry, Department of Psychiatry and Behavioural Sciences, Rawal Institute of Health Sciences, Rawal General and Dental Hospital, Lehtrar Road, Islamabad, Pakistan; 4 Dr. Nasir Ahmad Assistant Professor Centre for Education & Staff training University of Swat, Pakistan

**Keywords:** Anxiety and quality of life, Depression, Domestic violence

## Abstract

**Objectives:**

To find out the relationship of domestic violence with depression, anxiety and quality of life in married women in hospitals of Rawalpindi and Islamabad.

**Methods:**

This co-relational study was conducted in Rawalpindi Institute of Health Sciences from January 2019 to December 2019. All the females’ patients who were the victim of domestic violence were the population of the study. Consecutive non-probability sampling technique was used for selection of sampling from the target population. The inclusion criterion for this study was diagnosed case of domestic violence. DASS 21 (The Depression, Anxiety and Stress Scale) and Quality of life (WHO) scales were administered to 116 patients.

**Results:**

The study’s key results were that domestic abuse has positive relationship with depression, anxiety, and stress. It was also found that domestic abuse has a negative relationship with quality of life of those who have been subjected to domestic violence of this sort.

**Conclusion:**

It was concluded that domestic violence whether verbal, physical, emotional or sexual has strongly effects the mental health and quality of life of abused women.

## INTRODUCTION

Domestic violence is a grave combination of violent and abusive behavior that adults exhibit against their partners. Majority of the people perceive that physical abuse for example hitting, slapping, and beating between spouses as domestic violence.^1^ The Americans Overseas Domestic Violence Crisis Center explained five major types of abuse, which are physical, emotional, sexual, social, and spiritual abuse. Domestic violence exists in all ethnic and racial communities across the world and women are mostly among those who are often the victim of domestic violence.^2^

Women have a central role in family in any culture, and their mental, physical and social well-being is closely connected to society’s overall well-being. Health service providers around the globe are giving much importance to physical, mental and reproductive health of women for a healthy society. WHO report on violence and health illustrated that in most countries of the world women are mostly maltreated, and the major victims of domestic violence. It also found that women who were victimized by their partners had higher rates of depression, anxiety, and phobias than those who were not victimized.^3^ Unfortunately, Those Women who belonged to disadvantaged groups and are living poor life in different communities around the world are at high risk to become victim of domestic violence. Domestic violence can seriously damage the physical and mental health of abused women. It also undermines their social, economic, spiritual and emotional well being of the victimized women and even it can affect the whole society. It has been considered a major element that contributes to the poor health of women.^4^

The relationship of domestic abuse with mental health problems can be measured by the mental level of those women who were abused by their intimate partner and who are seeking treatment in mental health clinics. A research carried out in United States America revealed that women seeking treatment and help in family clinics, 20% of them had actually suffered from physical, emotional or sexual violence by their intimate partners.^5^ Mental health providers around the world are more concerned with increasing rate of mood disorders in women who are domestically abused. Studies conducted on domestic abuse and mental health of abused women found that common psychiatric disorders include depression, stress, post-traumatic stress disorder (PTSD), eating disorders, substance dependence, antisocial personality disorders, and non-affective psychoses.^6^ This has increased the burden on mental health facilities.

A study on spousal abuse among Pakistani women revealed that women had undergone a variety of harassment and abuse; these include physical assault, degradation, inhuman neglect, marginalization and poverty. This has severely influenced their social life, physical and mental wellbeing, mental states and relationships with other members of family and society.^7^ Another research on patriarchy and gender-based violence in Pakistan revealed that Thomson Reuter Foundation expert poll ranked Pakistan among top three dangerous countries for women in the world^8^. Bibi in Hyderabad, Sindh, Pakistan found that Domestic violence was quite common among married women.^9^ Hence there is need to investigate the relationship of domestic violence with depression, anxiety and quality of life of married women in Pakistan. Domestic violence as perceived by the women in this study was verbal violence (conflict, taunting, blaming and shouting), physical violence (beating, pushing, shoving, using any means such as hands, legs, sticks) and emotional violence ( feeling anxious or depressed due to conflicts with husband or in-laws). It is expected that the findings of this study will provide insight to the consequences of abuse experiences. The study has broad scope and long-term implications as paying attention to sufferings of abused women will ensure better psychological conditions for current and future generations of Pakistan.

## METHODS

This co-relational study was conducted in Rawalpindi Institute of Health Sciences. All the females’ patients who were the victim of domestic violence were the population of the study. Consecutive non-probability sampling technique was applied for selection of sampling from the population of the study. The inclusion criterion for this study was diagnosed cases of domestic violence who visited Rawalpindi Institute of Health Sciences for treatment. The study was approved by research ethics committee through Letter No. RISH-RCE/036/018 Dated: November 23, 2018. After the Approval by the research ethics committee, the study was carried out from January 2019 to December 2019. First the study was completely described to the subjects and then written informed consent was obtained. It was made clear that Subjects will not receive any money or other inducement for their participation. DASS 21 (The Depression, Anxiety and Stress Scale) and Quality of life (WHO) scales were administered to 116 patients.

## RESULTS

The demographic details are given in Table-I. The demographic characteristics included age, marital status, education, occupation and family setup of women who were exposed to domestic violence. The relationship of physical, emotional and sexual violence with anxiety, depression and stress is shown in Table-II while the relationship of physical, emotional and sexual violence with quality of life is shown in Table-III

**Table-I:** Demographic details.

Age		Frequency	Percent
	15-30	45	38.8
	31-45	59	50.9
	46 and above	12	10.3
Marital status	Married	80	69.0
	Divorced	26	22.4
	Widow	10	8.6
Education	Uneducated	10	8.6
	Primary	23	19.8
	Secondary	40	34.5
	Higher secondary	21	18.1
	Graduation and above	22	19.1
Occupation	House wife	69	59.5
	Working woman	47	40.5
Family setup	Joint	54	46.6
	Nuclear	62	53.4
N	116	116	100.0

**Table-II:** Co Relations between Domestic Violence and DASS.

		Anxiety	Depression	Stress
Domestic violence	Pearson correlation	0.424**	0.527**	0.441**
	Sig.	0.000	0.000	0.000
Domestic VP	Pearson correlation	0.584**	0.668**	0.482**
	Sig.	0.000	0.000	0.000
Domestic VE	Pearson correlation	0.380**	0.415**	0.274**
	Sig.	0.000	0.000	0.003
Domestic VS	Pearson correlation	0.449**	0.547**	0.393**
	Sig.	0.000	0.000	0.000

**Table-III:** Co Relations between Domestic Violence and Quality of Life.

		QOLMP	QOLRP	QOLSC	QOLPD	QOLR
Domestic VV	Pearson correlation	-0.578**	-0.526**	-0.406**	-0.403**	-0.295**
	Sig.	0.000	0.000	0.000	0.000	0.001
Domestic VP	Pearson correlation	-0.556**	-0.438**	-0.412**	-0.378**	-0.279**
	Sig	0.000	0.000	0.000	0.000	0.002
Domestic VE	Pearson correlation	-0.579**	-0.472**	-0.480**	-0.399**	-0.331**
	Sig.	0.000	0.000	0.000	0.000	0.000
Domestic VS	Pearson correlation	-0.572**	-0.447**	-0.443**	-0.394**	-0.387**
	Sig.	0.000	0.000	0.000	0.000	0.000

## DISCUSSION

Gender-based violence is a persistent global issue that significantly contributes to poor mental health of women across diverse cultures in the globe. Existing research studies elaborates that physical and psychological effects are common in women abused by intimate partner. This study found a strong positive relationship between verbal, physical, emotional and sexual domestic violence with anxiety, depression and stress and also found that domestic violence has negative relationship with quality of life. The finding of this study that verbal, physical, emotional and sexual domestic violence has strong positive relationship with anxiety, depression and stress is consistent with other studies. Hussain found that in Gilgit-Baltistan, Pakistan married women reported higher levels of domestic violence (psychological, physical & sexual) and lower mental health.^10^ The findings was also confirmed by Bibi S et al. who found that that in Hyderabad, Sindh, Pakistan, Domestic violence was quite common among married women.^9^ A study by Ferrari found that Women who recently experienced high level of domestic abuse and survive developed high level of depression, anxiety, and especially PTSD.^11^ Another study in Tehran, Iran conducted by Ahmadzad-Asl M et al. found that DV as a social factor is significantly correlated factor with depression and anxiety.^12^ This finding of our study is also consistent with Lacey, Nathanson Klingspohn and Duran.^13^-^16^

Domestic violence (IPV) also leads to serious physical harm, sexual abuse and problems with mental wellbeing. To create strategies that can dramatically enhance the quality of life of survivors and deter potential violence, it is important to understand the healthcare patterns of the victims. The finding of this study that domestic violence has negative relationship with quality of life is consistent with the findings of Lucena^17^, who found that those women who suffered from domestic violence has lower quality of life index (59.62) than those women who do not suffered from domestic violence has high quality of life index (66.80). Another study found a significant negative relationship between the severity of psychological, physical, sexual injuries of domestic violence and quality of life.^18^ The finding is also consistent with Tavares, and Tavoli.^17^,^19^

This research demonstrates that it is beneficial to use methods to address the problem of domestic violence against women, to connect it with mental health and with quality of life, to encourage discussion, to generate new ideas and prospects for the future.

### Limitations of the study

This research was limited to only one tertiary hospital of Rawalpindi, which is a small sample. The use of large sample and cross-sectional research design would improve the results of future studies.

## CONCLUSION

Domestic violence whether verbal, physical, emotional or sexual has strongly effects the mental health and quality of life of abused women.

### Authors’ Contribution:

**MM:** Conception and design

**MUG & NM:** Collection and assembly of data.

**NM & NA:** Analysis and interpretation of the data.

**MM, NM & NA:** Drafting of the article.

**MM & MUG:** Critical revision of the article for important intellectual content.

**NM & NA:** Statistical expertise.

**MM:** Final approval and guarantor of the article and accountable for the study.

## References

[1] Yick AG, Agbayani-Siewert P (1997). Perceptions of domestic violence in a Chinese American community. J Interpers Violence.

[2] Feroz U, Jami H, Masood S (2015). Role of early exposure to domestic violence in display of aggression among university students. Pak J Psychol Res.

[3] Garcia-Moreno C, Jansen HA, Ellsberg M, Heise L, Watts C (2005). WHO multi-country study on women's health and domestic violence against women. Geneva:World Health Organization.

[4] Kaur R, Garg S (2008). Addressing domestic violence against women:An unfinished agenda. Indian J Community Med.

[5] Coker AL, Smith PH, McKeown RE, King MJ (2000). Frequency and correlates of intimate partner violence by type:physical sexual, and psychological battering. Am J Public Health.

[6] Campbell JC (2002). Health consequences of intimate partner violence. Lancet.

[7] Sultan H, Khawaja AO, Kousir T (2016). Spousal abuse among Pakistani women:A thematic analysis. Pak J Clin Psychol.

[8] Hadi A (2017). Patriarchy and gender-based violence in Pakistan. Eur J Soc Sci Educ Res.

[9] Bibi S, Ashfaq S, Shaikh F, Qureshi PMA (2014). Prevalence instigating factors and help seeking behavior of physical domestic violence among married women of Hyderabad Sindh. Pak J Med Sci.

[10] Hussain H, Hussain S, Zahra S, Hussain T (2020). Prevalence and risk factors of domestic violence and its impacts on women's mental health in Gilgit-Baltistan Pakistan. Pak J Med Sci.

[11] Ferrari G, Agnew-Davies R, Bailey J, Howard L, Howarth E, Peters TJ, Sardinha L (2016). Domestic violence and mental health:a cross-sectional survey of women seeking help from domestic violence support services. Glob Health Action.

[12] Ahmadzad-Asl M, Davoudi F, Zarei N, Mohammad-Sadeghi H, Rasoulian M (2016). Domestic violence against women as a risk factor for depressive and anxiety disorders:findings from domestic violence household survey in Tehran Iran. Arch Womens Ment Health.

[13] Lacey KK, McPherson MD, Samuel PS, Powell Sears K, Head D (2013). The impact of different types of intimate partner violence on the mental and physical health of women in different ethnic groups. J Interpers Violence.

[14] Nathanson AM, Shorey RC, Tirone V, Rhatigan DL (2012). The prevalence of mental health disorders in a community sample of female victims of intimate partner violence. Partner Abuse.

[15] Klingspohn DM (2018). The importance of culture in addressing domestic violence for First Nation's women. Fron Psychol.

[16] Duran S, Eraslan ST (2019). Violence against women:Affecting factors and coping methods for women. JPMA.

[17] Lucena KD, Vianna RP, Nascimento JA, Campos HF, Oliveira EC (2017). Association between domestic violence and women's quality of life. Rev Lat-Am Enferm.

[18] Asadi S, Mirghafourvand M, Yavarikia P, Mohammad-Alizadeh-Charandabi S, Nikan F (2017). Domestic violence and its relationship with quality of life in Iranian women of reproductive age. J Family Violence.

[19] Tavoli Z, Tavoli A, Amirpour R, Hosseini R, Montazeri A (2016). Quality of life in women who were exposed to domestic violence during pregnancy. BMC Pregnancy and Childbirth.

